# Serum metabolomics analysis revealed metabolic disorders in Parkinson’s disease

**DOI:** 10.1097/MD.0000000000033715

**Published:** 2023-06-09

**Authors:** Tian-Tian Lan, Le Chang, Li-Wei Hou, Zhen-Zhen Wang, Dong-Chu Li, Zi-Han Ren, Tao Gu, Jian-Wen Wang, Gui-Sheng Chen

**Affiliations:** a Clinical Medical College of Ningxia Medical University, Yinchuan, China; b People’s Hospital of Ningxia Hui Autonomous Region, Yinchuan, China; c Department of Neurology, Ningxia Medical University General Hospital, Yinchuan, China; d Cranial Laboratory of Ningxia Medical University, Yinchuan, China.

**Keywords:** lipids and lipid-like molecules, metabolomics, Parkinson’s disease, sphingolipid metabolic pathway

## Abstract

Parkinson’s disease (PD) is by now the second of the most prevalent neurodegenerative diseases in the world, and its incidence is increasing rapidly as the global population ages, with 14.2 million PD patients expected worldwide by 2040.

**Methods:**

We gathered a completion of 45 serum samples, including 15 of healthy controls and 30 from the PD group. We used non-targeted metabolomics analysis based on liquid chromatography-mass spectrometry to identify the molecular changes in PD patients, and conducted bioinformatics analysis on this basis to explore the possible pathogenesis of PD.

**Results:**

We found significant metabolomics changes in the levels of 30 metabolites in PD patients compared with healthy controls.

**Conclusion:**

Lipids and lipid-like molecules accounted for the majority of the 30 differentially expressed metabolites. Also, pathway enrichment analysis showed significant enrichment in sphingolipid metabolic pathway. These assessments can improve our perception on the underlying mechanism of PD as well as facilitate a better targeting on therapeutic interventions.

## 1. Introduction

Parkinson’s disease (PD) that is a progressive neurodegenerative disorder featured by motor and non-motor syndromes, which include resting tremor, stiffness, bradykinesia, postural instability, constipation and depression, ultimately leading to disability.^[1]^ PD is by now the second of the most prevalent neurodegenerative diseases worldwide as well as impacting millions of people, predominantly those older than 65 years of age.^[1]^ The incidence of PD is increasing rapidly as the global population ages. The dominant pathological signature in PD are a substantial degeneration and reduction of dopaminergic neurons from the substantia nigra pars compacts.^[2]^ The pathogenic mechanisms underlying PD has not been fully disclosed, and curative therapies are currently not available. The treatment of PD includes drug therapy, surgical treatment, botulinum toxin therapy, exercise therapy, psychological intervention, nursing care and so on. Drug therapy is the first choice and the main treatment in the whole treatment process. Levodopa is the standard treatment for PD and the most effective symptomatic drug in the drug treatment of PD. The treatment of PD, whether drugs or surgery, can only improve the symptoms, can’t stop the development of the disease, let alone can’t be cured. Several pathogenic pathways have been identified, including defective mitophagy, lysosomal mechanisms and accumulation of aberrant or misfolded proteins.^[2]^ The most reliable basis for the diagnosis of PD is the pathological sections obtained by postmortem examination, which is impossible for the patient before death. Currently, the diagnostics of PD is mainly on the basis of its clinical manifestations. Specific mainly according to the age of onset, hidden onset, and slow progress of the 3 major symptoms – quiescent tremor, muscle stiffness, and bradykinesia. Nevertheless, to even have a rigorous clinical diagnosis of PD conducted by specialists in movement disorders, they have a predictive value of less than 90 percent for late-stage disease, and this predictive value is much worse in the very early stages of the disease.^[3]^ In addition to pharmacological treatment, there is a growing interest in early intervention. Unable to accurately identify PD before motor symptoms develop can lead to loss of scarce intervention time.^[4]^ Therefore, there is an urgent need to develop reliable markers for the diagnosis of PD and for predicting its prognosis. In further, the establishment of PD-specific biomarkers may help reveal new steps in pathogenesis, detect the disease at an early stage, indicate the severity of the disease, and provide insight into disease mechanisms that can in turn be used to identify new therapeutic targets for effective drugs.

Additional information suggests that changes, involving metabolic disorders, may precipitate as well as facilitate neurodegeneration. Many metabolic pathways associated with PD are disturbed, including the metabolism of catecholamines, tryptophan, caffeine, xanthine, hypoxanthine, kynurenine, purines, amino acids, and fatty acids.^[5]^ Levels of some amino acids, fatty acids and redox metabolites have been identified as altered through the blood of individuals suffering from PD. Metabolomics technology has been a useful instrument to describe alterations of metabolites in human body fluids. Already known to analyze variability in metabolic pathways across a wide range of diseases, which include neurodegenerative diseases.^[6]^ This approach not only studies numerous pathways and simultaneously quantifies small molecules and their metabolites, but also provides high sample definition and accelerated analysis speed.^[6]^ Blood is an excellent specimen for the detection of biomarkers as well as for clinically diagnostic purposes due to the fact that it is straightforward to obtain, comparatively noninvasive, and highly amenable to analysis. In this study, we determine metabolic biomarkers and pathogenic pathways of PD patients by investigating the metabolite changes associated with PD using an LC-MS-based untargeted metabolomics method by assaying the levels of sera metabolites in PD patients.^[7]^

## 2. Materials and methods

### 2.1. Clinical data

Serum specimens were received from 30 patients who were suffering from PD in the neurology outpatient clinic of the General Hospital of Ningxia Medical University. Inclusion criteria were as follows: PD diagnosis in accordance with the European Federation of Neurology Recommendations for Social and Motor Disorders Association European section; patients taking only dopamine analogues or dopamine agonists.^[8]^ Patients should be excluded if the following exclusion criteria are considered: secondary PD or Parkinson’s syndrome; severe systemic disease; stroke, brain surgery, Alzheimer’s disease, motor neuron disease, or any other medical history central nervous system disease.^[8]^ All included PD patients were screened by experienced neurologists. Sex- and age-matched 15 healthy controls (HC) were randomly recruited from the medical screening center. Except for PD, all subjects were free of chronic renal failure, systemic infections, malignancy, cardiac or hepatic dysfunction, autoimmune disease, stroke, or neurodegenerative disease.^[9]^ A series of 45 serum specimens were obtained, 15 from the HC group and 30 from the PD group. The female/male ratio was 7/8 on the control team, and 15/15 on the PD team. A thorough description of the participants is provided in Table 1. Blood was collected from all 45 participants in the morning before the patients ate or drank. Serum specimens were maintained at −80°C continuously up to liquid chromatograph-mass spectrometer (LC-MS) analysis. This research was endorsed by the Ethics Committee of the General Hospital of Ningxia Medical University and was undertaken in conformance with the Declaration of Helsinki. All of the involved participants had provided written known agreement prior to enrollment in this research.

**Table 1:** Information table of differential metabolites in PD group.

Differential metabolite name	Super class	Up/down
p-Aminobenzoic acid	Benzenoids	Up
Cinnamyl phenylacetate	Benzenoids	Up
Vignatic acid B	Organic acids and derivatives	Up
2-Dodecylbenzenesulfonic acid	Benzenoids	Up
Armillarivin	Lipids and lipid-like molecules	Up
Nummularine B	Organic acids and derivatives	Up
Ganoderic acid L	Lipids and lipid-like molecules	Up
5-Nonyltetrahydro-2-oxo-3-furancarboxylic acid	Organoheterocyclic compounds	Up
Allopurinol-1-ribonucleoside	Nucleosides, nucleotides and analogues	Up
Salfredin B11	Organoheterocyclic compounds	Up
Lithocholate 3-O-glucuronide	Lipids and lipid-like molecules	Up
N-Oleoylethanolamine	Organic nitrogen compounds	Up
trans-Ferulic acid	Phenylpropanoids and polyketides	Up
Piceatannol 4’-galloylglucoside	Phenylpropanoids and polyketides	Up
3-Methoxytyrosine	Organic acids and derivatives	Up
DGTS(4:0/22:6)	Lipids and lipid-like molecules	Down
TAG(14:3/14:3/14:3)	Lipids and lipid-like molecules	Down
Cholesterol glucuronide	Lipids and lipid-like molecules	Down
Crassostrea Secocarotenoid	Lipids and lipid-like molecules	Down
TAG(12:3/20:6/20:6)	Lipids and lipid-like molecules	Down
Plastoquinone 3	Lipids and lipid-like molecules	Down
PS(20:5(5Z,8Z,11Z,14Z,17Z)/20:0)	Lipids and lipid-like molecules	Down
PA(14:1(9Z)/18:4(6Z,9Z,12Z,15Z))	Lipids and lipid-like molecules	Down
Ginsenoside F2	Lipids and lipid-like molecules	Down
Saringosterol 3-glucoside	Lipids and lipid-like molecules	Down
PI(16:1(9Z)/16:1(9Z))	Lipids and lipid-like molecules	Down
PS(20:2(11Z,14Z)/22:6(4Z,7Z,10Z,13Z,16Z,19Z))	Lipids and lipid-like molecules	Down
5-Butyl-2-ethyloxazole	Organoheterocyclic compounds	Down
3’-(2’“,3”‘-Digalloylglucosyl)-phloroacetophenone	Nucleosides, nucleotides and analogues	Down
1-hexadecyl-glycero-3-phosphate	Lipids and lipid-like molecules	Down

Down = metabolite levels were down-regulated in the PD group, PD = Parkinson’s disease, Up = metabolite levels were up-regulated in the PD group.

### 2.2. Derivation of metabolites

Samples were first shifted to eppendorf tubes, vortexed for 30 seconds with the addition of extracts and processed in an ice water bath for 10 minutes. This was followed by incubation at −40°C for 1 hour to obtain the sedimented proteins. Finally, centrifugation is performed at 4°C for 15 minutes. The derived supernatant was shifted to a new glass vial for further analysis. Quality control (QC) samples were made by combining equal amounts of supernatant from all samples together.^[10]^

### 2.3. LC-MS/MS analyses

LC-MS/MS analyses were performed using an UHPLC system (Vanquish; Thermo Fisher Scientific, Jiading District, Shanghai, China) with a UPLC HSS T3 column (2.1 mm × 100 mm, 1.8 μm) coupled to Orbitrap Exploris 120 mass spectrometer (Orbitrap MS; Thermo). The mobile phase consisted of 5 mmol/L ammonium acetate and 5 mmol/L acetic acid in water (A) and acetonitrile (B). The auto-sampler temperature was 4 ℃, and the injection volume was 2 μL. The Orbitrap Exploris 120 mass spectrometer was used for its ability to acquire MS/MS spectra on information-dependent acquisition mode in the control of the acquisition software (Xcalibur; Thermo). In this mode, the acquisition software continuously evaluates the full scan MS spectrum. The ESI source conditions were set as following: sheath gas flow rate as 50 Arb, Aux gas flow rate as 15 Arb, capillary temperature 320 ℃, full MS resolution as 60,000, MS/MS resolution as 15,000 collision energy as 10/30/60 in NCE mode, spray Voltage as 3.8 kV (positive) or −3.4 kV (negative), respectively.^[11]^

### 2.4. Data pre-processing and evaluation

Raw data were converted to the mzXML version using ProteoWizard and an in-house program written in R and based on XCMS was used for peak detection, extraction, alignment and integration. Afterwards, metabolite annotation was performed using an in-house MS2 database (BiotreeDB).^[12]^ The cutoff point for annotation was set to 0.3.^[13]^

### 2.5. Pre-processing of raw data and statistical analysis

For the positive ion model, the raw data included 6 quality control samples together with 45 laboratory samples, from which 13,757 peaks were retrieved. In order to reduce the impact of errors in the assay system on the results and to make the results better highlight the biological significance, we conducted a number of procedures to prepare and organize the raw data.^[14]^ It mainly includes the following steps: deviation value filtering (Filtering a single Peak to remove noise. Filtering the deviation values based on the relative standard deviation); Missing value filtering (Filtering on a single Peak. Retaining only peak area data with a single set of nulls of not more than 50% or with nulls of not more than 50% for all groups); Missing value padding (Missing values in the raw data were simulated. Numerical simulation method for filling the minimum value of 1/2); Data standardization processing (Normalization by internal standard). After processing, 9632 Peak were retained. Then, single-factor and multi-factor statistical analysis was performed on the qualitative and quantitative results of metabolites to screen out the differential metabolites, and a series of bioinformatics analyses were performed to visually display and explore the biological functions of the differential metabolites.^[15]^

## 3. Results

We have collected a total of 45 serum samples, 15 from HC and 30 from the PD group. The female/male ratio was 7/8 for the HC team and 15/15 for the PD team. There was no remarkable difference from the HC team and PD team with regard to gender composition. This demonstrates that subjects in the HC team and PD team are relatively comparable.

**Figure 1.**
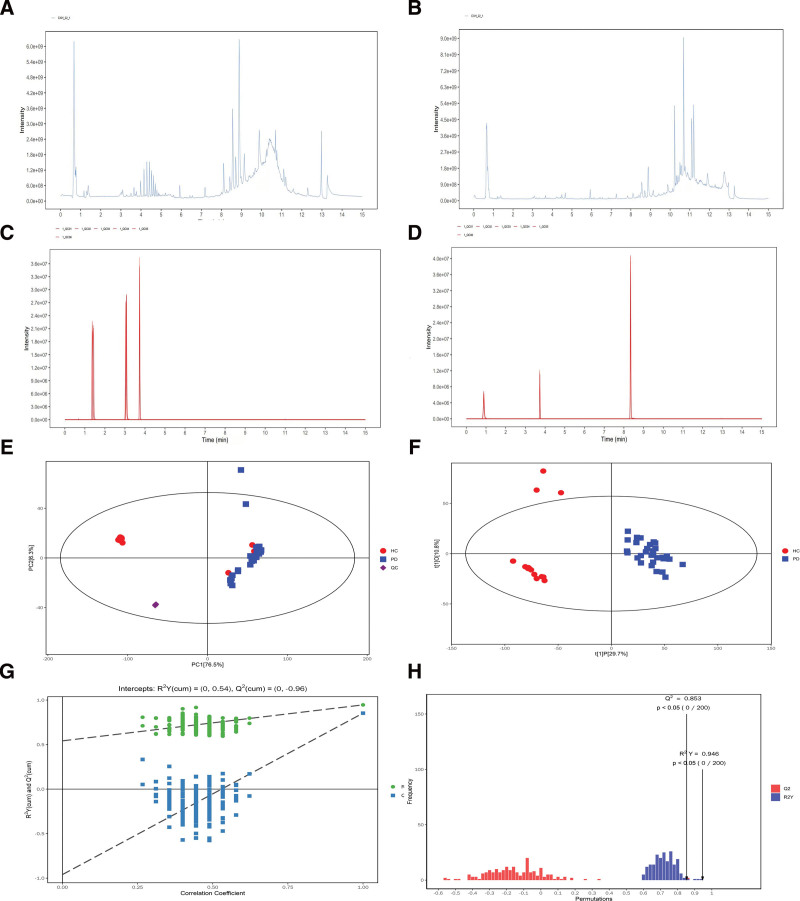
(A) TIC diagram of positive ion mode for UHPLC-OE-MS detection of QC samples. (B) TIC diagram of negative ion mode for UHPLC-OE-MS detection of QC samples. (C) EIC diagram of internal standard positive ions in all QC samples. (D) Internal labeling of negative ions in all QC samples EIC graph. (E) Scatterplot of PCA scores for all samples (including QC samples). The horizontal and vertical coordinates in the figure indicate the scores of the first and second ranked principal components, respectively. (F) Scatterplot of OPLS-DA model scores for PD group versus HC group. The plot’s horizontal coordinates represent the first fundamental component’s projected principal component scores, indicating the between-sample group differences. The orthogonal principal component scores are indicated by the vertical coordinates, indicating the within-sample group differences. (G) A dot plot of the PD group’s displacement test results versus the HC group’s OPLS-DA model. (H) Replacement test results for the PD group versus the OPLS-DA model for the HC group histogram. EIC = extracted ion chromatogram, HC = healthy controls, OPLS-DA = orthogonal partial least squares-discriminant analysis, PCA = principal component analysis, PD = Parkinson’s disease, QC = quality control, TIC = total ion current, UHPLC-OE-MS = ultra-high performance liquid chromatography-orbittrap explois mass spectrometer.

To verify the robustness of the system we examined QC sample from the original data using the positive and negative ion patterns, respectively. We overlaid the total ion chromatograms of the QC samples for comparison and obtained total ion current plots of the positive and negative ion modes of the QC samples (Fig. 1A and B). The overlapping of response intensities and retention times of the QC samples were good as seen in the plots, demonstrating the strong stability of the approach.

During the detecting phase, it’s critical to keep an eye on the instrument’s stability and signal normalization in real time. The retention time and response intensity stability of the internal standard in QC samples can be estimated by comparing the response peak height of the internal standard between QC samples. Comparing all QC samples in the internal standard positive and negative ions extracted ion chromatogram graph can illustrate the internal standard’s retention time and response intensity stability in QC samples (Fig. 1C and D). This demonstrates that the data acquisition stability of the instrument is fairly good.

These results indicate that the methodology is scientifically acceptable in terms of reproducibility and stability. The considerable variations between the 2 groups discovered using multivariate statistical analysis are more likely the result of genuine metabolite changes than of technological errors.^[14]^

The scatter plot of PCA scores obtained by principal component analysis can effectively highlight the overall distribution trend of metabolomics data. The scatter plot of principal component analysis scores (Fig. 1E) for the full sample (including the QC sample) shows that our sample is within the 95% confidence interval.^[16]^

Then, for non-orthogonal and orthogonal variables analysis, we used the statistical approach of orthogonal partial least squares-discriminant analysis (OPLS-DA) to filter out orthogonal variables in metabolites that were not related with categorical variables. As a result, more trustworthy information on metabolite group differences in terms of the degree of correlation between experimental groups can be obtained. The 2 groups are very significantly differed, as revealed in the OPLS-DA score plot (Fig. 1F), and the samples are essentially in the 95 percent confidence interval. It suggests that the metabolites in the PD group have changed significantly.

To avoid testing for overfitting and to examine the statistical significance of the model, we employed a permutation test to measure its quality. The replacement test results for the OPLS-DA model (Fig. 1G and H) show that the original model is resilient and there is no embeddedness.

**Figure 2.**
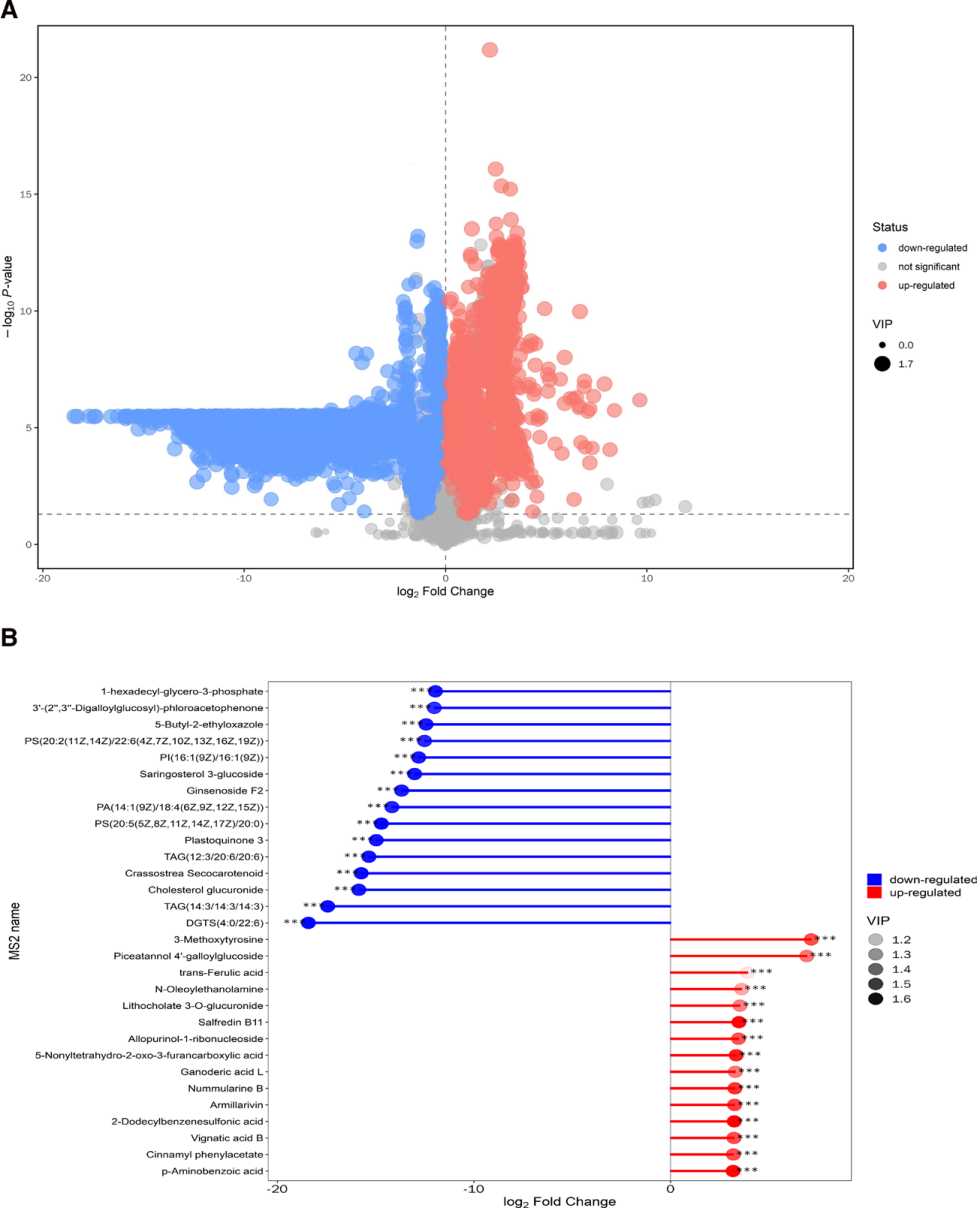
(A) Volcano plot for group PD versus HC. A peak is illustrated by each point on the volcanic diagram. Each substance’s multiplicative change is depicted by the horizontal coordinate, the vertical coordinate by the *P* value, and the scatter size by the VIP value. (B) For the PD versus HC group, a matchstick analysis was performed. The log-transformed change multiples are represented by the horizontal coordinates in the graph, the names of the differentiating metabolites in the PD group are represented by the vertical coordinates, and the color hues of the dots show the VIP value size. (*Represents significance *.01 < *P* < .05, **.001 < *P* < .01, **P < .001.) HC = healthy controls, PD = Parkinson’s disease, VIP = Variable Importance in the Projection.

To filter differential metabolites in this study, the *P* value of the Student’s *t* test (*P* value) and the Variable Importance in the Projection (VIP) of the first principal component of the OPLS-DA model were utilized. To be considered as differential metabolites in our investigation, the 2 criterion of VIP more than 1 and *P* value less than .05 were to be satisfied together. Because volcano plots can show the general distribution of metabolite differences across groups, we used them to show the findings of our screening for differential metabolites. The following are the results of the PD group versus the HC group (Fig. 2A): Significantly raised metabolites are displayed in red, substantially downregulated metabolites are shown in blue, and metabolites that are not substantially different are highlighted in gray.

The VIP scores of metabolites were positively correlated with the contribution to sample classification, with higher VIP values resulting in more reliable separation between groups. Therefore, metabolites with high VIP score rankings are more noteworthy. Using a logarithmic transformation with a base of 2, we determined the corresponding ratios of the numerical variables of the differentiated metabolites. After that, 30 differential metabolites with a significant degree of variation were chosen based on the log-transformed fold of variation and the magnitude of VIP values (Fig. 2B). The information table provides the names and classification information of these 30 different metabolites (Table 1). Fifteen of these differential metabolites had elevated levels in the PD group, including p-aminobenzoic acid, cinnamyl phenylacetate, 2-dodecylbenzenesulfonic acid, vignatic acid B, nummularine B, 3-methoxytyrosine, ganoderic acid L, 5-nonyltetrahydro-2-oxo-3-furancarboxylic acid, salfredin B11, lithocholate 3-O-glucuronide, N-oleoylethanolamine, trans-ferulic acid, and piceatannol 4’-galloylglucoside, among others. There were 15 differential metabolites with reduced levels in the PD group, including PI(16:1(9Z)/16:1(9Z)), 1-hexadecyl-glycero-3-phosphate, PS(20:5(5Z,8Z,11Z,14Z,17Z)/20:0), PA(14:1(9Z)/18:4(6Z,9Z,12Z,15Z)), saringosterol 3-glucoside, cholesterol glucuronide, ginsenoside F2, crassostrea secocarotenoid, plastoquinone 3, 5-butyl-2-ethyloxazole, and 3’-(2’“,3”‘-digalloylglucosyl)-phloroacetophenone, among others. Classification of these significantly altered differential metabolites revealed major changes in benzenoids, lipids and lipid-like molecules, organic acids and derivatives, organic nitrogen compounds, organic oxygen compounds, organoheterocyclic compounds, organooxygen compounds, phenylpropanoids, polyketides, nucleosides, nucleotides, and analogues.

**Figure 3.**
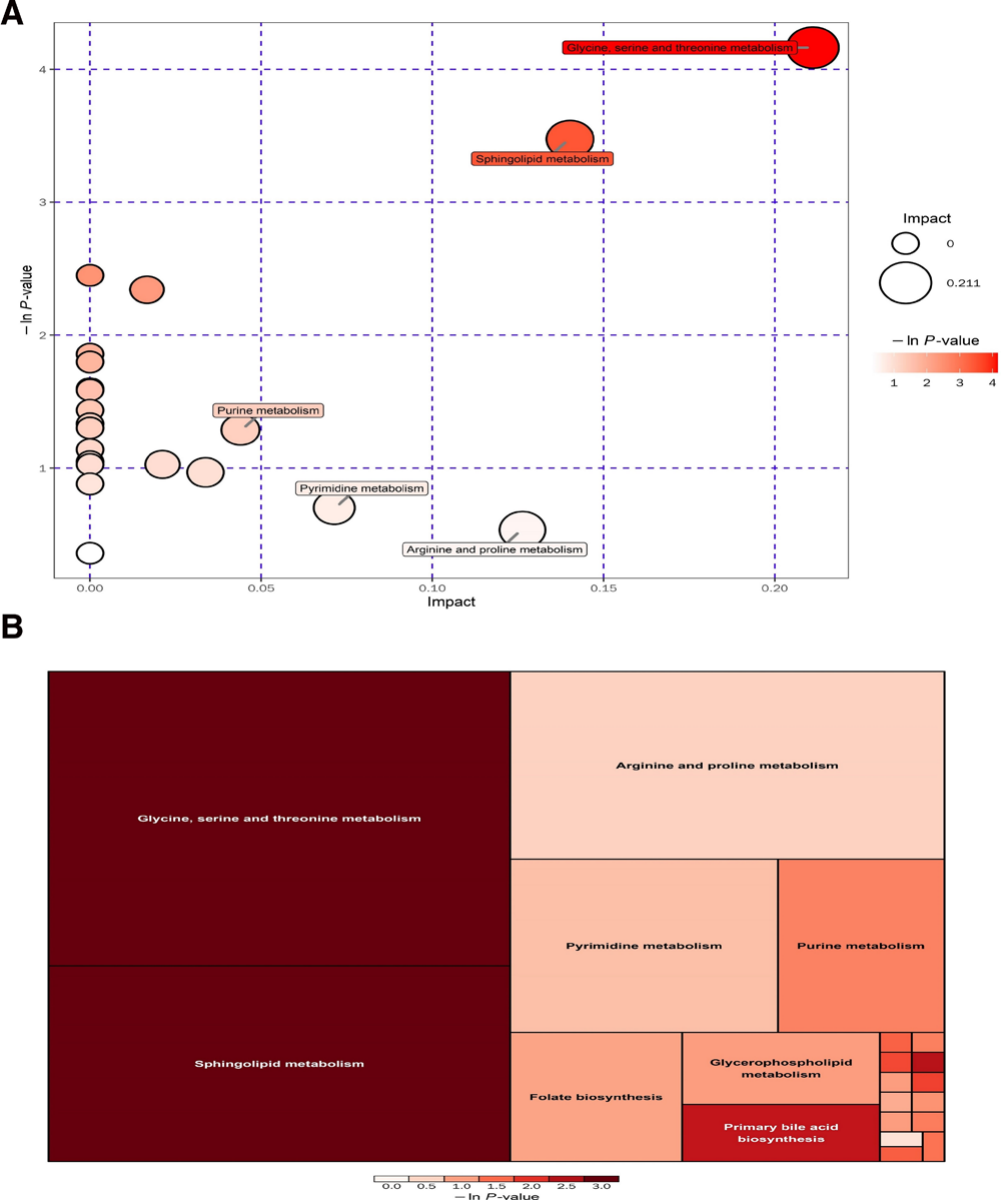
(A) Graphical route analysis bubble diagram for the PD versus HC groups. Every bubble symbolizes a metabolic route, with the horizontal coordinate indicating the magnitude of the pathway’s impact factor and the vertical coordinate indicating the width of the enrichment analyses’ *P* value. Following research are generally focused on the darker and bigger bubbles with a higher degree of enrichment in the route. (B) Pathway analysis in a rectangular tree diagram for the PD versus HC groups. Each square represents a metabolic route, with the diameter of the triangle indicating the magnitude of the pathway’s effect factor and the color indicating the enrichment analyses’ *P* value. Following research are generally focused on large dark squares with a high degree of enrichment. HC = healthy controls, PD = Parkinson’s disease.

The KEGG online database is the most widely used metabolic pathway database for metabolic network research, and it’s used in metabolic pathway enrichment analysis to find the most relevant metabolic pathways and probable processes. We filtered the metabolic pathways with the strongest association to the differential metabolites in the PD group using a comprehensive assessment of the metabolic pathways where the differential metabolites are detected (including pathway enrichment analysis and topology analysis). The findings of the metabolic pathway enrichment research are shown as bubble plots (Fig. 3). The quantity of the pathway’s impact factor in the topological analysis is indicated by the horizontal coordinate of each bubble in the bubble diagram; the bigger the bubble, the stronger the influence factor. The *P* value of the enrichment analysis is represented by the vertical coordinate of the bubble placement and the color of the bubble; the smaller the *P* value and the more important the enrichment, the deeper the color of the bubble. As a consequence, we decided to focus our research on dark-colored big bubbles with high enrichment and strong effect factors after analyzing enrichment analysis and topological analysis. As shown in Figure 3, metabolic dysregulation of glycine, serine, and threonine metabolism, sphingolipid metabolism, purine metabolism, pyrimidine metabolism, arginine and proline metabolism, primary bile acid biosynthesis, folate biosynthesis, and glycerophospholipid metabolism may play a significant role in the pathogenesis of PD.

## 4. Discussion

PD is a widespread neurological illness that is expected to affect 14.2 million people globally by 2040. The buildup of Lewy bodies and the dramatic loss of dopaminergic neurons in the dense section of the substantia nigra are the hallmark clinical alterations, although the specific pathogenic process is unknown.^[17]^ Consequently, there is an urgent need for biomarkers for PD diagnosis and better understanding of disease pathogenesis.

At the same time, blood, as an easily accessible and information-rich biofluid for patients, has become a convenient tool for exploring the pathogenesis of many neuropsychiatric disorders. Consequently, we employed LC-MS-based untargeted metabolomics analysis to identify molecular changes in PD patients, and also conducted bioinformatics analysis on this basis to exploit the possible pathogenesis of PD. By extensively examining serum levels of metabolites in PD patients, we found profound metabolomic alterations in levels of 30 metabolites in PD patients compared to normal controls, with significant reductions in 15 metabolites, including 1-hexadecyl-glycero-3-phosphate, saringosterol 3-glucoside, cholesterol glucuronide, ginsenoside F2, crassostrea secocarotenoid, plastoquinone 3, 5-butyl-2-ethyloxazole, and 3’-(2’“,3”“-digalloylglucosyl)-phloroacetophenone, among others. A total of 15 metabolites were significantly increased, including p-aminobenzoic acid, cinnamyl phenylacetate, 2-dodecylbenzenesulfonic acid, vignatic acid B, nummularine B, 3-methoxytyrosine, ganoderic acid L, 5-nonyltetrahydro-2-oxo-3-furancarboxylic acid, salfredin B11, lithocholate 3-O-glucuronide, N-oleoylethanolamine, trans-ferulic acid, and piceatannol 4”-galloylglucoside, among others.

The significant differential metabolites we found in PD patients fall into the following major categories, including benzenoids (benzene and substituted derivatives), lipids and lipid-like molecules (glycerophospholipids, steroids and steroid derivatives, prenol lipids), organic acids and derivatives (carboxylic acids and derivatives), organic nitrogen compounds, organic oxygen compounds, organoheterocyclic compounds (lactones), phenylpropanoids and polyketides (cinnamic acids and derivatives). These significantly altered differential metabolites involved glycine, serine and threonine metabolism, sphingolipid metabolism, purine metabolism, pyrimidine metabolism, arginine and proline metabolism, primary bile acid biosynthesis, folate biosynthesis and glycerophospholipid metabolism. The bulk of the 30 differentially elevated metabolites found by this non-targeted metabolomics investigation were lipids and lipid-like compounds. Lipids and lipid-like molecules can be further classified into: sphingolipids, glycerophospholipids, lipid acylates, steroids and their derivatives, etc. Also, pathway enrichment analysis showed significant enrichment in sphingolipid metabolic pathway, which indicates that sphingolipid metabolic pathway is activated in PD patients.

Sphingolipids are a class of lipids that contain a sphingolipid substrate and a group of aliphatic amino alcohols. Sphingolipids are common components of cell membranes, and they play a vital role in regulating and integrating cellular signals. Ceramide, ceramide-1-phosphate, glucosyl ceramide, lactosyl ceramide, galactosyl ceramide, sphingosine, sphingosine galactoside and sphingosine-1-phosphate in the sphingolipid metabolic pathway are not only inactive precursors of sphingolipid metabolism, but also important effector molecules in cell signaling. Sphingolipids and some metabolites are involved in many important signaling processes that regulate cell growth, differentiation, aging and programmed cell death, resulting in a variety of biological functions.^[18]^

Lipid is an important component of cells, which undertakes the functions of transmitting information and transporting substances in biological activities.^[19]^ Studies have shown that there are multiple links between lipid droplets and neurodegenerative diseases. For example, the abnormal accumulation of lipid droplets in neurons can induce α synuclein to be transformed into a form that prevents protein hydrolysis, thus causing the accumulation of α synuclein in human neurons, suggesting that lipids may play a role in the etiology of PD.^[20]^

In conclusion, this metabolomic analysis revealed that PD is associated with dyslipidemia and that activation of sphingolipid metabolic pathways may be a potential pathogenesis of PD, which may provide a new therapeutic approach to modify PD. These findings warrant further research to explore the potential of sphingolipids as PD biomarkers and therapeutic targets. Limitations of this research are that some unknown interactions of drugs or other factors may also lead to metabolic differences between groups, and other relevant factors need to be excluded in the future. It is unclear whether the identified metabolites are specific to PD, and in future studies, we need further validation in patients with PD to further study the specific biomarkers of PD.

## 5. Conclusion

To summarize, our findings suggest that glycine, serine, and threonine metabolism, sphingolipid metabolism, purine metabolism, pyrimidine metabolism, arginine and proline metabolism, primary bile acid biosynthesis, folate biosynthesis, and glycerophospholipid metabolism may all play a significant role in PD pathogenesis. Metabolic disorders of sphingolipid metabolism were highlighted as possible key metabolic events in PD. These assessments may improve our understanding of PD pathogenesis and facilitate target screening for therapeutic interventions.

## Acknowledgements

Special thanks to Dr Xiaoqing Zhuang for her support and advice on this study. Thanks also to Ningxia Medical University General Hospital for supporting this research.

## Author contributions

**Conceptualization:** Tian-Tian Lan, Le Chang, Li-Wei Hou, Zhen-Zhen Wang, Dong-Chu Li, Zi-Han Ren, Gui-Sheng Chen.

**Data curation:** Tian-Tian Lan, Le Chang, Li-Wei Hou, Zhen-Zhen Wang, Dong-Chu Li, Zi-Han Ren, Gui-Sheng Chen.

**Formal analysis:** Tian-Tian Lan, Le Chang, Li-Wei Hou, Zhen-Zhen Wang, Gui-Sheng Chen.

**Funding acquisition:** Tian-Tian Lan, Le Chang, Li-Wei Hou, Zhen-Zhen Wang, Gui-Sheng Chen.

**Investigation:** Zhen-Zhen Wang, Tao Gu, Jian-Wen Wang.

**Methodology:** Tian-Tian Lan, Le Chang.

**Project administration:** Tian-Tian Lan, Gui-Sheng Chen.

**Resources:** Tian-Tian Lan, Le Chang, Li-Wei Hou.

**Software:** Dong-Chu Li, Zi-Han Ren.

**Supervision:** Dong-Chu Li, Zi-Han Ren, Tao Gu, Jian-Wen Wang.

**Visualization:** Tian-Tian Lan, Zhen-Zhen Wang, Dong-Chu Li, Gui-Sheng Chen.

**Writing – original draft:** Tian-Tian Lan.

**Writing – review & editing:** Tian-Tian Lan, Gui-Sheng Chen.
